# Some Remarks on Chloroform

**Published:** 1880-02-20

**Authors:** A. Hazlewood


### *SOME REMARKS ON CHLOROFORM.*

BY A. HAZLEWOOD, M. D.

Without desiring to disparage the value of sulphuric ether as an anæsthetic agent, or to enter the arena as a controversialist, I think a few words will not be amiss in confirmation of the already large volume of testimony favorable to chloroform, as the anæsthetic *par excellence.* The boon of anæsthesia is too great a relief to suffering humanity to have any opposition among medical practitioners, no matter to which of the many claimants the supremacy as its discoverer is due. I believe no one contests the claim of Sir J. Y. Simipson as the discoverer and introducer of chloroform, as *the* anæsthetic for all serious operations. It is unnecessary here to enter into the details of its introduction to the profession; the curious in such matters will find interesting and entertaining reading concerning this part of the subject in the works published by Sir J. Y. Simpson. In these days much controversy has been and is indulged in concerning the relative merits of sulphuric ether and chloroform as anæsthizing agents; and that the interest in the matter is considerable, is evidenced by the second edition of Dr. Turnbull's manual being so soon called for. That Dr T. has striven to give a candid and unbiased opinion in the matter, I do not doubt; nor am I prepared yet to state dogmatically, that I disagree with his conclusions, viz., that ether is the safer anæsthetic of the two. But I do think that the evident and disagreeable objections to ether, so well recognized and understood by Dr. Simpson, which were the incentive to induce him to undertake the experiments culminating in the discovery of chloroform, have not lessened or been overcome to this day. The objections are, briefly:

The large quantity required to produce anæsthesia.

Its tardy and frequently incomplete action; in other words, its unreliability.

The prolonged and often distressing stage of excitement preceding full anæsthesia, entailing much loss of valuable time.

The very disagreeable sensations experienced by the person taking ether.

The expense from requiring so large a quantity.

The unpleasant perfume or odor, and the pertinacity with which said odor clings to the clothing of every one present when administered, and is also exhaled subsequently from the lungs of the patient.

The persistent nausea induced, often continuing days after administration.

Its bulkiness and the necessity for a special inhaler.

Its inflammability, its specific gravity being but .720; boiling point under a pressure of 30 inches, 98° Fah.

These are weighty objections, requiring many precautions to lessen or avoid, without, as I consider, any counterbalancing advantages over chloroform, which is at the same time free from all these objections. But the main point is yet to be considered, viz., the safety to the individual inhaling.

The state or condition known as anæsthesia is not a natural one; it is induced by several agents, cold, alcohol and several other substances, among which chloroform and sulphuric ether are most widely known and used, when the anæsthetic condition is required to be produced artificially.

As, therefore, we have a pathological condition, produced by our own volition, we are in duty bound to use the best agent, and with the best accessories known, to insure, as far as practicable, the best results to our patients.

Which shall we use, then, is the question, when it is desirable to produce artificial anæsthesia, chloroform or sulphuric ether? I prefer chloroform mostly, always with children, and for the majority of purposes with adults also. Why do I do so? Chloroform is agreeable in its odor to most persons; requires but a small quantity; has a short stage of delirium preceding anæsthesia; is not inflammable, and requires no special apparatus for its administration; is more manageable and reliable; seldom produces much vomiting when proper care is exercised in preparing patient; and I think is equally safe in the hands of a competent administrator.

The plea made for sulphuric ether as the safer of the two is, that fewer deaths follow its administration than happens with chloroform. This and this alone. Supposing this statement to be true, does it follow that the chloroform is at fault? Are any of us prepared to give up morphia as a therapeutic agent, simply because more persons die from its abuse or ignorant use than from paregoric? Or are we ready to discard the obstetric forceps, because, forsooth, some ignoramus has so abused his office as to irremediably destroy some poor suffering woman? Will you not answer at once, no! but insist on a greater amount of experience and knowledge in the use of such potent remedies for good or ill.

How often is this requisition ignored? How many of the deaths attributed to chloroform can be justly laid to ignorance or carelessness, or to the impurity of the drug used?

My belief is, *very many*. Do I err when I affirm that in many operations the task of giving chloroform or ether is intrusted either to a non-professional or to a tyro in the ranks? The remark is made that any one can give the anæsthetic, but the efficient help is required to assist the operator. And when efficient help is scarce, how can it be otherwise?

The careful practitioner hardly dares give veratrum or hydrocyanic acid without careful personal watching, and yet it is not unusual to alter the condition of existence by permitting a deadly poison to be inhaled, without equally careful supervision.

I claim that the physician, administering an anæsthetic, has an equally responsible position with the surgeon performing the operation. On his coolness, adroitness and knowledge depends largely the success of the operation. When the anæsthetic is in competent hands the surgeon has less anxiety, his mind is free to be exclusively engaged with his special manipulations. On the contrary, when he has the further anxiety of how the anæsthetic is at work, his mind is apt to be confused, the operation prolonged, and the operation more unsatisfactory. The physician assigned the duty of administering the anæsthetic must ignore all else; his attention is needed absolutely and without intermission to regulating and observing its action. Yet, how often is it otherwise. The patient's face is covered with a towel saturated with chloroform, his breathing stertorous, helpless to avert danger, and all those who have him under their charge entirely engrossed in the steps of the operation! No wonder death occurs, but I do not blame the chloroform. The only wonder is that there are so few deaths under such circumstances. Chloroform can be, and is frequently, used for several hours at a time, so that the patient is entirely helpless, is truly anæsthetized, and yet neither stertorous nor irregular breathing nor disturbance of the heart's action is produced. In the very large majority of cases it can always be so. The idiosyncrasy that cannot tolerate chloroform I believe to be very rare.

Heavy stertor is evidence of too much interference with the respiratory function being produced, and is a condition beyond the anæsthesia required for painless operations. The instructions of Dr. T. Hughes, as given in the London *Lancet*, are concise and to the point. He writes: "If I were about to be placed under the influence of chloroform, I would say, never mind my pulse, never mind my heart; leave my pupil to itself; keep your eye on my breathing; and, if it becomes embarrassed to any extent, take an artery forceps and pull my tongue well out." It is claimed that the late Mr. Syme faithfully observed this rule and never lost a case although he had administered chloroform 5,000 times. I do not claim so extended an experience, although I have given it many times during the past 20 years, and I, also, have never seen a fatal case from its use. Chisholm claims to have administered it at least once a day for many years, and during 25 years given it 6,000 times, and has never seen a fatal case. He further says: "I have accepted Syme's axiom, and given chloroform to every one, regardless of visceral complications, who has applied to me for a serious surgical operation, and I have yet to see the first death, either in my own practice or that of my friends."

Of the many deaths ascribed to chloroform, 370 in all so far reported, a careful sifting would undoubtedly very largely diminish the number properly to be attributed to this drug. The proportion of deaths to inhalations is estimated as 1 to 20,000. Even if this number be correct, which I much doubt, the percentage is very small, smaller, indeed, than opium has to be charged with; and when we strike out those cases in which vomiting has lodged pieces of food in the larynx, or where the system has been overcharged with the vapor, the cases showing an idiosyncrasy will be found reduced to a very small number indeed. If the list of deaths ascribed to chloroform published in Dr. Turnbull's manual be examined, a very large number will be found where no autopsy was made, especially of the larynx; nor history given of the method of administration. Under such circumstances, it is unfair to charge the chloroform itself with the death unless the possibility of careless administration, or closure of the larynx from vomited matter, has been excluded.

The points, then, in my argument are briefly:

That as an anæsthetic, chloroform has, undoubtedly, many advantages over sulphuric ether.

That in the hands of a competent administrator it is equally safe with sulphuric ether.

That an anæsthetic, when given, should be under the charge of a competent physician or expert; that he should be responsible for that department, as much so as the surgeon operating is in his sphere; and paid a fee therefor commensurate with his ability and time occupied. And to those who may be called upon to perform this duty, I would emphasize the instructions of those writers and experts who have made a study of anæsthesia, to-wit: Quietness in room, and silence of the bystanders; a stomach not too long empty, about four hours since an ordinary meal preferred; loose clothing; recumbent posture (the position on stomach is sometimes dangerous from interference with movements of respiration; sitting up is even worse, tending to produce anæmia of the brain and syncope). The patient should swallow a small quantity of good distilled liquor before commencing the inhalation.

The inhalation should be at first a large admixture of atmospheric air and gradually increased as the system becomes accustomed, until anæsthesia is perfect, when its further use must be governed by the special circumstances of the case.

The nitrite of amyl and ammonia should be on hand to counteract any bad tendency that may arise. The caution cannot be too often emphasized, never give an anæsthetic to ladies without a third party being present; nor to expect so pleasant an action when actions upon the genitals or toes are to be made.—*Detroit Lancet.*

